# Entrapment within an ottoman storage bed: an unusual accidental asphyxial death

**DOI:** 10.1007/s12024-022-00473-6

**Published:** 2022-03-23

**Authors:** Alessandro Cinquetti, Giorgia Franchetti, Giulia Fichera, Chiara Giraudo, Guido Viel, Giovanni Cecchetto

**Affiliations:** 1 Legal Medicine, Department of Cardiac, Thoracic, Vascular Sciences and Public Health, University of Padova, Via Falloppio 50, 35121 Padova, Italy; 2 Pediatric Radiology Unit, Padova University Hospital, Via Giustiniani 2, 35121 Padova, Italy; 3 Unit of Advanced Clinical and Translational Imaging, Department of Medicine – DIMED, Via Giustiniani 2, 35121 Padova, Italy

**Keywords:** Forensic pathology, Forensic histology, Mechanical asphyxia, Accidental death, Postural asphyxia

## Abstract

Herein, we present an uncommon forensic case of death by asphyxia. The victim was a woman whose body at death scene investigation (DSI) was discovered beside an ottoman storage bed. According to the rescue team, who had moved the body before our arrival, the body was originally found in the prone position and stuck with the neck, thorax and arms within the bed. Examination of the body showed hypostasis that was mainly distributed to the face and the lower chest while sparing the neck and the upper chest. The face was markedly swollen, and the eyes were congested with blood. Dissection and histology revealed pulmonary oedema and emphysema of both lungs. Integrating circumstantial, radiology and autopsy data, it was established that the victim, while trapped between the mattress and the edge of the ottoman storage bed, died by mechanical asphyxia due to cervical-thoracic compression and postural asphyxia acting simultaneously.

## Introduction

“Asphyxia” or “asphyxiation” is defined as an oxygen deficit caused by specific traumas resulting in hypoxic-ischaemic brain damage and death. These traumas include various mechanisms, such as strangulation, obstruction of respiratory movements and obstruction of the respiratory orifices and/or the airways [1]. Asphyxia also occurs as strangulation of elderly or psychotic persons who become trapped in bed [2]. Bibliographic research of cases of death by asphyxia in association with the term “bed” refers mainly to paediatric cases of suffocation and/or sudden infant death syndrome (SIDS) or cases of elderly individuals with neurologic disorders. Scientific papers describing cases of purely forensic interest mention cases of traumatic compression by bed rails or foldaway beds [3–5]. In this article, we present a singular case in which the victim was trapped between the mattress and the edge of an ottoman storage bed. Circumstantial data and postmortem findings are discussed herein to assess the pathophysiological mechanisms of death.

## Case report

### Death scene investigation

**Fig. 1:**
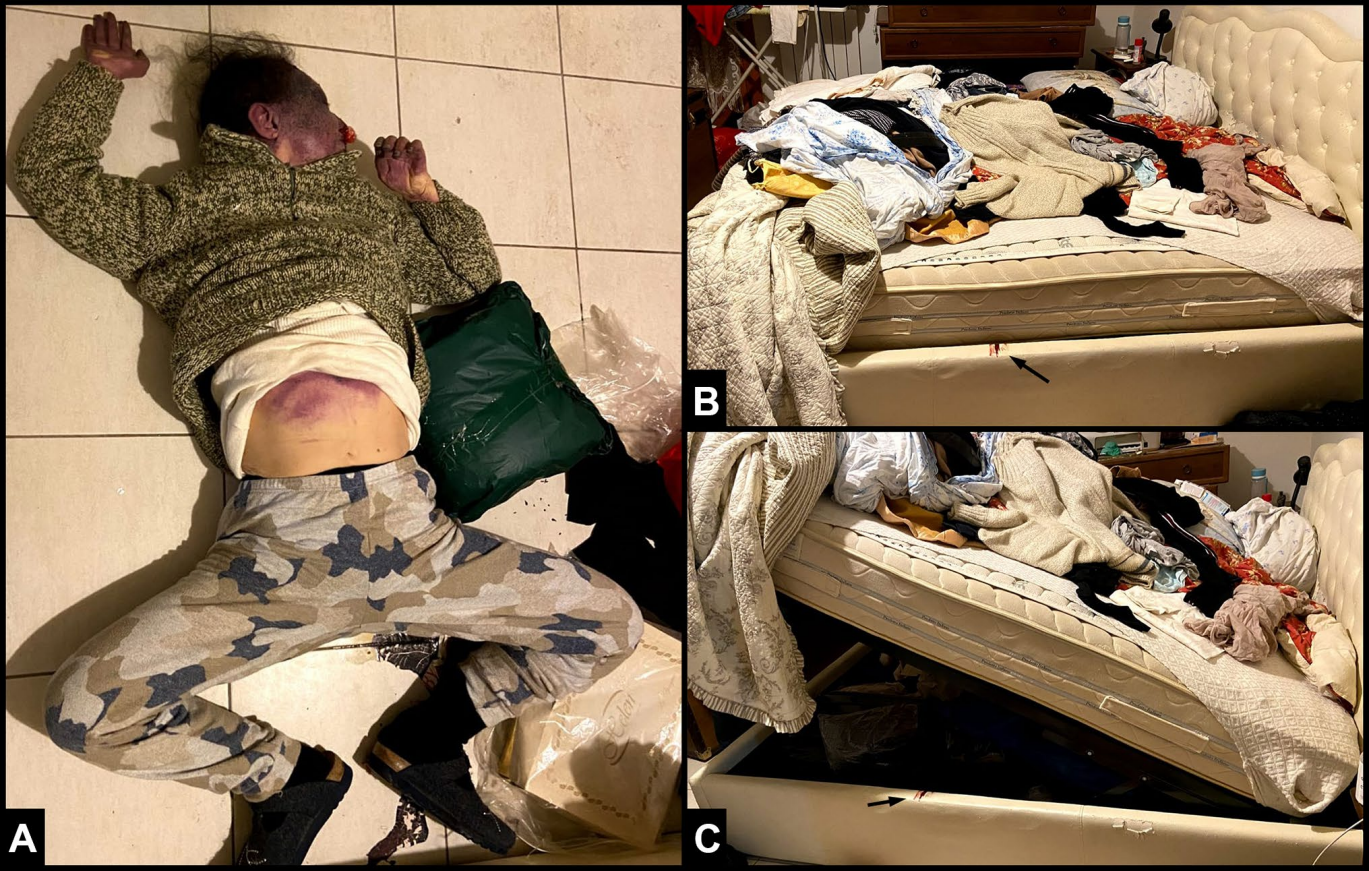
At DSI, the body, which had been removed from its original location by the rescue team, was lying supine on the floor near the bed (**A**). The mattress was cluttered with clothes and blankets (**B**–**C**); at inspection, traces of blood were found on the lateral edge of the container (**B**–**C**, black arrow)

The victim was a 56-year-old woman whose body was found by the rescue team to be in the prone position and trapped inside an ottoman storage bed, which consisted of a mechanism that lifted the bed base and mattress to reveal a storage space beneath them. Upon our arrival, the body had already been removed from its original location for rescue purposes and was lying supine on the floor near the bed, with the head rotated towards the left side, the arms abducted, the forearms flexed, and the lower limbs abducted and flexed at the knees (Fig. 1A). Examination of the body revealed fixed purple hypostases dotted with petechiae located on the face, chest and upper abdominal quadrants, with a pale rectangular-shaped area extending from one arm to the other, including the anterior surface of the neck and bilateral clavicular regions. Fixed hypostasis was also distributed on the anterior surface of the upper limbs and the palms. Another area spared from hypostasis was recognizable at both wrists. Rigor mortis involved the jaw and both upper and lower limbs. The body temperature was equal to the environmental temperature. Inspection of the bed revealed traces of blood smearing the lateral edge of the container below the bed (Fig. 1B, C, black arrow). The mattress was cluttered with clothes and blankets (Fig. 1B, C). The bed suspension mechanism was defective, as it was not equipped with the system designed to hold up the mattress. At room inspection, several packages of antidepressants and benzodiazepines were found.

### Forensic autopsy

**Fig. 2:**
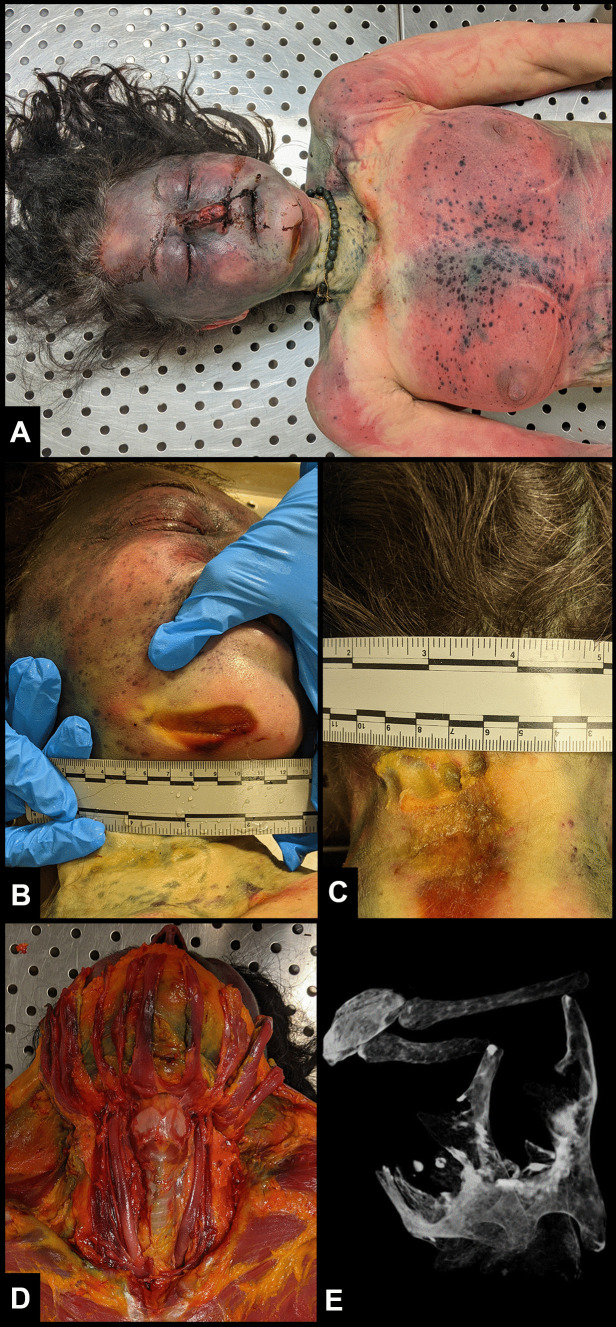
At autopsy, the distribution of postmortem lividities was comparable to that found at the time of death (**A**). An oval-shaped abrasion, with a size of 4.5 × 2.5 cm, was noticed under the chin on the right side (**B**). On the posterior cervical region, an oval-shaped abrasion with a length of 5 cm and a width of 3 cm was present (**C**). Upon anterior and posterior dissection of the neck, the soft tissues corresponding to the above-described abrasions were infiltrated with blood, while the cervical vertebrae were not fractured. Both jugular veins were dilated (**D**). Micro-CT scans showed no dislocations or fractures of the larynx and the hyoid bone (**E**)

A forensic autopsy was performed 3 days after death was pronounced. The deceased individual was 150 cm in height and 55 kg in weight. At autopsy, the distribution of postmortem lividities was comparable to that found at the death scene investigation (DSI) (Fig. 2A). The conjunctivae were completely engorged with blood, the face was swollen and congested, and the tongue protruded between the dental arches. Mouth and nose bleeding and cyanosis of the face and both hands were observed. An oval-shaped abrasion, with a size of 4.5 × 2.5 cm, was noticed under the chin on the right side (Fig. 2B). On the posterior cervical region, an oval-shaped abrasion with a length of 5 cm and a width of 3 cm was detected (Fig. 2C). Upon internal examination, although diffuse haemorrhagic infiltration of the scalp over the frontal and bilateral temporoparietal regions was noticed, neither fractures of the cranial bones nor encephalic lesions were identified. Upon anterior and posterior dissection of the neck, the soft tissues corresponding to the above-described abrasions were infiltrated with blood, while the cervical vertebrae were not fractured. Both jugular veins were dilated (Fig. 2D). The lungs were markedly congested and overinflated. Hyoid bone and larynx specimens were collected to perform a micro-computed tomography (micro-CT) analysis, which is useful to detect any potential fractures or microfractures.

### Histological and toxicological analysis

Histological examination of the lungs displayed acute pulmonary emphysema with a predominantly subpleural disposition; in other areas of the pulmonary parenchyma, atelectasis, intra-alveolar oedema and vascular congestion were observed. Other histological findings were chronic bronchitis and macrophages within the alveolar spaces. Toxicological analyses in blood revealed subtherapeutic concentrations of diazepam, flurazepam and escitalopram, while no ethanol or drugs of abuse were detected.

### Micro-computed tomography analysis

The larynx and hyoid bone were scanned using a micro-CT 1275 device (Skyscan, Bruker, Kontich, Belgium; 15.7 µm isotropic voxel size, 83 kV, 120 µA, exposition time 6050 ms, rotation step 0.7, frame averaging 2, 1280 × 1024 pixel field of view). CTVox (Skyscan, Belgium) was used to elaborate the 3D volumetric reconstructions. The micro-CT scan showed no dislocations or fractures of the larynx or the hyoid bone (Fig. 2E).

## Discussion

Based on external (massive congestion and oedema of the face, cyanosis of the hands) and internal findings (acute emphysema, oedema and vascular congestion of the lungs), death was attributed to asphyxia [6–8]. The body temperature, fixation of hypostases and rigor mortis placed the time of death beyond 24 h before the discovery of the body.

**Fig. 3:**
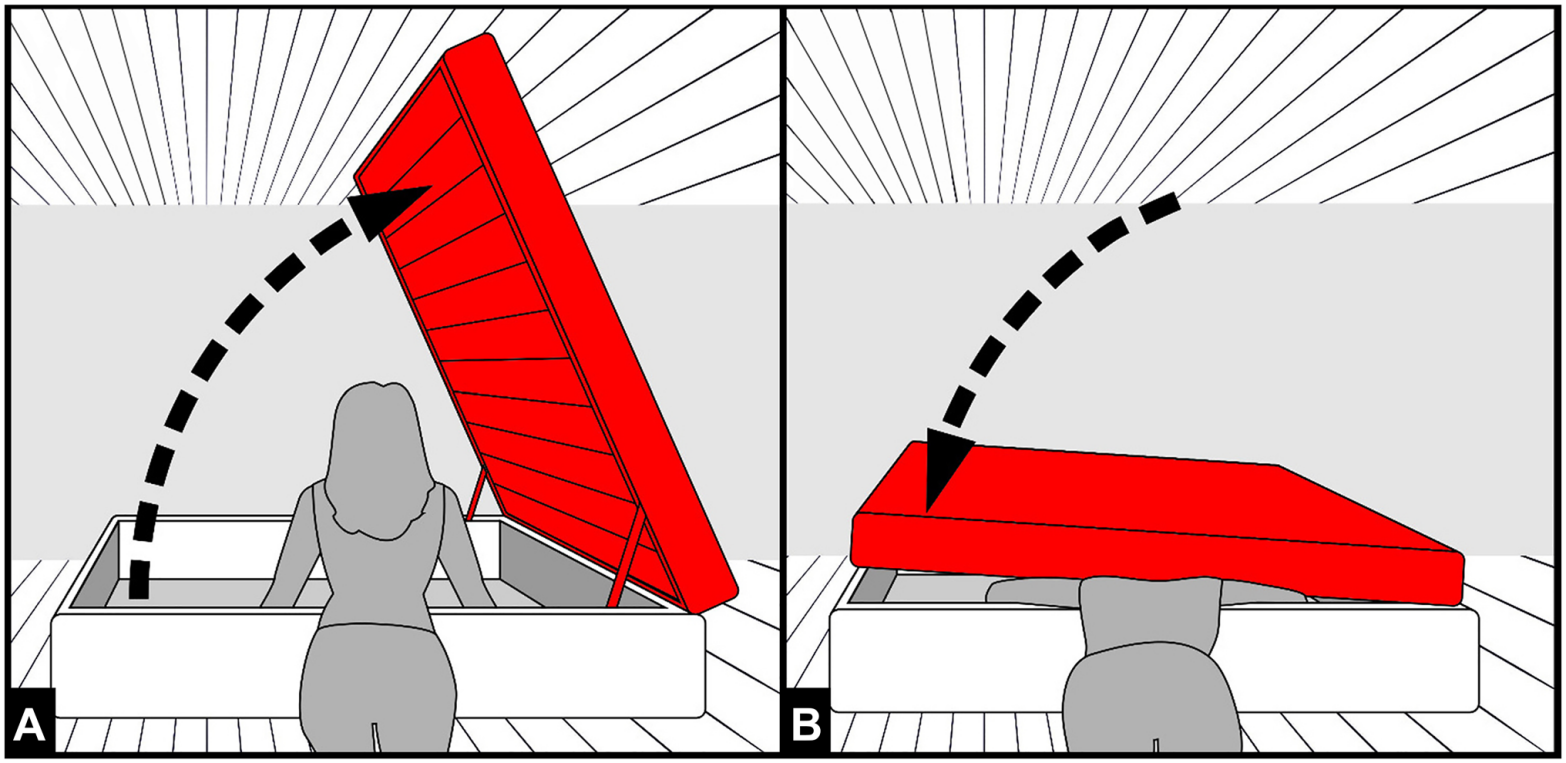
Sketch depicting the most likely mechanism of compression of the neck between the left bed frame and the partially lifted bed base and mattress (**A**–**B**)

Regarding the position of the victim at the time of death (before being moved by the rescue team), the distribution of postmortem lividities (face, chest and upper limbs without affecting the abdomen) was suggestive of a prone posture, with the face and trunk placed lower than the abdomen [9]. On the other hand, the sparing of the transverse band from hypostasis, the dilatation of jugular veins, the presence of an abrasion in the posterior cervical region and haemorrhages of the neck muscles were indicative of compression of the neck between the left bed frame and the partially lifted bed base and mattress (Fig. 3A–D), causing airway occlusion and obliteration of the common carotids and the jugular veins [10, 11]. The hypostasis-free areas located on both wrists also suggested that the hands leaned on the edge of the storage compartment. Furthermore, with the application of a high-resolution radiological technique, such as micro-CT [12], which is useful to identify microfractures, we could additionally examine the upper airway without any risk of iatrogenic damage occurring during dissection, excluding injuries to the horns of the larynx or the hyoid bone, usually detected in cases of manual strangulation [13–15]; considering the abovementioned autopsy findings, indeed, compression of the neck seemed highly probable [16]. Cervical compression could have induced a loss of consciousness mediated by carotid baroreceptors causing bradycardia and reduction of arterial flow to the brain [17, 18].

The absence of hypostases in the upper part of the chest (i.e., bilateral supraclavicular regions) and the high intensity of asphyxia signs (i.e., petechial haemorrhages in the skin, sclera and conjunctiva) indicated a compression mechanism (i.e., traumatic asphyxia), with inadequate and ineffective respiratory movements of the rib cage [6]. Moreover, the peculiar position of the victim, trapped between the bed frame and the bed base/mattress, not only hindered breathing but also affected circulation due to the impaired venous return and the pressure on the diaphragm exerted by the abdominal viscera, determining postural/positional asphyxia [9, 19–21].

Integrating all the above-described findings (DSI, autopsy, radiology and histology), it was concluded that the woman’s death was caused by asphyxiation by three different mechanisms acting simultaneously: neck compression and traumatic and postural asphyxia.

Over time, given the great variety of pathophysiological mechanisms underlying asphyxiations, several classifications have been proposed in the forensic literature. Recently, Sauvageau and Boghossian, to simplify and standardize the above classification as much as possible, proposed four main categories based on autoptic and circumstantial findings: suffocation, strangulation, mechanical asphyxia and drowning [22]. This classification does not explicitly contain the so-called atypical neck compression (i.e. compression not caused by a ligature, by strangling or by hanging) [4]. Moreover, the proposed definition of mechanical asphyxia refers to the restriction of respiratory movements, either by the position of the body (postural or positional asphyxia) or by external chest compression by a heavy object (traumatic asphyxia).

Thus, based on Sauvageau and Boghossian, the case reported and discussed here should be classified as “traumatic asphyxia”, with no mention of the adjunctive asphyxia mechanism due to “neck compression”.

Commenting on the classification proposed by Sauvageau and Boghossian, Byard proposed an alternative focused on the pathophysiological processes that interfere with oxygen transfer and/or transport [23–25], identifying 5 different classes.

1. *Failure to supply* adequate amounts of oxygen that occurs when there is a critical reduction in environmental oxygen levels due to displacement of oxygen by other gases or to consumption of oxygen without replacement.
2. *Failure of transfer* that takes place in cases of obstruction or the presence of an obstacle in the air passages from the nose and mouth to the alveoli. This condition may occur due to internal or external obstruction of the airways or even because of compromise of the thoracic cage function.
3. Reduced/absent blood flow to the brain leads to *failure of transport* of oxygen to the tissues, a condition that occurs as a result of the compression of the neck vessels.
4. *Failure of uptake and utilization of oxygen* occurs in the case of cyanide poisoning because cells are unable to function metabolically despite an oxygen-rich milieu.
5. C*ombination of the other mechanisms*, as happens in non-judicial hangings where vascular occlusion, vagal inhibition, and airway compression and occlusion coexist simultaneously.

We retain that the present case could be fully classified into the last category of Byard’s proposal, where the failure to transfer oxygen from the environment to the lungs coexists with the inability to efficiently transport oxygen to the brain and other tissues due to the compression of the arterial and venous vessels of the neck. The vascular compression not only contributed to death but also to the loss of consciousness. This information, in addition to the other circumstantial (position of the victim, malfunction of the suspension system of the ottoman storage bed), autopsy-related (no signs of violence or grasping on the body), radiological (no signs of manual or ligature strangulation), and toxicological data (low blood concentration of benzodiazepines) support an accidental manner of death, which is most common in traumatic and positional asphyxial fatalities [26, 27].

In conclusion, although both the above-commented classifications are widely disseminated and accepted throughout the forensic community, we believe that, in complex and unusual cases, identifying and classifying the exact pathophysiological mechanisms of asphyxia (as suggested by Byard) could add valuable information to the reconstruction of the dynamics of the fatal event.

Last but not least, the position of the victim and the loss of consciousness undoubtedly facilitated the impossibility of wriggling her body and lifting the bed. For this reason, we believe that the storage space of ottoman beds should be preferably emptied or loaded under the supervision of another person, although we recognize that this could be unpractical. Indeed, in the case reported here, a second person at the scene would have promptly lifted the bed and applied first aid, probably preventing the death of the victim. We also underline that the safety systems must be periodically reviewed by qualified personnel to detect and repair defects and damage that can put people’s lives at risk. Finally, the localization of the bloodstain on the lateral edge of the bed defined the distance of the body from the bed suspension fulcrum. We recommend that the instruction manuals for ottoman storage beds should recommend loading/unloading the storage space by positioning oneself on the side opposite to the lever fulcrum. Thus, although this would probably require a non-ordinary effort, in the event of a malfunction of the suspension system, the person would be in an advantageous lever position to lift the bed base and mattress.

## Key points

1. A 56-year-old woman was found dead entrapped within an ottoman storage bed.
2. The cause of death was a traumatic and postural asphyxia combined with neck compression.
3. The main international classifications of mechanical asphyxia are critically discussed.
4. Ottoman beds should preferably be loaded under the supervision of another person, and their safety systems should be periodically reviewed by qualified personnel.
